# Roadmap to Innovation of HTA Methods (IHTAM): insights from three case studies of quantitative methods

**DOI:** 10.1017/S0266462324000564

**Published:** 2024-11-05

**Authors:** Li Jiu, Junfeng Wang, Jan-Willem Versteeg, Yingying Zhang, Lifang Liu, Francisco Javier Somolinos-Simón, Jose Tapia-Galisteo, Gema García-Sáez, Milou A. Hogervorst, Xinyu Li, Aukje K. Mantel-Teeuwisse, Wim G. Goettsch

**Affiliations:** 1 Division of Pharmacoepidemiology and Clinical Pharmacology, Utrecht Institute for Pharmaceutical Sciences, Utrecht University, Utrecht, The Netherlands; 2 Centre for Health Economics, University of York, York, UK; 3 The European Organisation for Research and Treatment of Cancer, Brussels, Belgium; 4 Bioengineering and Telemedicine Group, Centro de Tecnología Biomédica, ETSI de Telecomunicación, Universidad Politécnica de Madrid, Madrid, Spain; 5 CIBER-BBN: Networking Research Centre for Bioengineering, Biomaterials and Nanomedicine, Madrid, Spain; 6 Groningen Research Institute of Pharmacy, Faculty of Science and Engineering, University of Groningen, Groningen, The Netherlands; 7 National Health Care Institute, Diemen, The Netherlands

**Keywords:** conceptual framework, innovation, health technology assessment, method, case study

## Abstract

**Objectives:**

A conceptual framework, called Innovation of Health Technology Assessment Methods (IHTAM), has been developed to facilitate the understanding of how to innovate methods of health technology assessment (HTA). However, the framework applicability has not been evaluated in practice. Hence, we aimed to explore framework applicability in three cases of method innovation that are part of the HTx project and to develop a roadmap to improve framework applicability.

**Methods:**

The IHTAM framework was applied to three cases of innovating HTA methods. We collected feedback from case study leaders and consortium members after a training session, an approximately 1-year follow-up of periodic case study meetings, and a general assembly meeting where innovation progresses of the three cases were reported through surveys and interviews. Feedback was then summarized using an open-coding technique.

**Results:**

According to feedback, the framework provided a structured way of deliberation and helped to improve collaboration among HTA stakeholders. However, framework applicability could be improved if it was complemented by a roadmap with a loop structure to provide tailored guidance for different cases, and with items to elaborate actions to be taken by stakeholders. Accordingly, a 48-item roadmap was developed.

**Conclusions:**

The IHTAM framework was generally applicable to the three case studies. A roadmap, with loop structure and actionable items, could complement the framework, and may provide HTA stakeholders with tailored guidance on developing new methods. To further examine the framework applicability, we recommend stakeholders to apply the IHTAM framework and its roadmap in future practice.

## Introduction

Methods of health technology assessment (HTA) refer to methods relevant to the full scope of an HTA process (1;2). According to the HTA Core Model from the European network for HTA, the HTA scope can be categorized into nine domains, including but not limited to clinical effectiveness, costs and economic evaluation, and patient and social aspects (3). Also, according to the European Patients’ Academy on Therapeutic Innovation, an HTA process generally has three phases: collecting and reviewing scientific evidence of a health technology, making decisions on reimbursement and pricing, and implementing decisions and monitoring impact (4). Therefore, the term “HTA methods” has broad implications with a large number of examples. One example is the measurement of patient-reported outcomes, through which patient aspects are considered during the collection of evidence, such as quality of life (5). Another example is the use of decision-analytic models for health economic evaluation, which investigates clinical effectiveness and costs for HTA decision-making (6).

HTA methods may be repeatedly developed and implemented, in other words, innovated, for multiple reasons. One reason is the emergence of novel health technologies to which traditional HTA methods may not be suited. For example, complex health technologies, which include combinations of health technologies, personalized treatment, and treatment pathways, pose requirements for novel methods that support more tailored decision-making (7). Another reason is the changed availability of data that could be used for HTA. For example, the increasing use of real-world data (RWD) poses challenges on data quality and creates needs for methods to assess the quality of data sources (e.g., data registry) or studies using RWD (8). In addition, the variety of HTA settings (e.g., developed vs. developing countries) creates barriers to transferring an existing HTA method in one setting to another, and creates needs for improving existing methods or developing a new method in the local setting (9;10).

While Innovation of HTA Methods (IHTAM) is often needed, HTA stakeholders, such as clinicians, policymakers, patient associations, third-party payers, and healthcare industry (11), often lack a general understanding on how to innovate HTA methods, and how they could engage in the innovation process. To facilitate such understanding, a conceptual framework, called IHTAM, has been developed under the umbrella of large H2020 project, HTx, that is focused on the development of new HTA methods (1). The IHTAM framework was developed in two stages. First, concepts of innovating HTA methods were identified and synthesized in two scoping reviews, one on the current practice of innovating methods, that is, existing HTA frameworks, and the other on the theoretical foundations for innovating methods outside the HTA discipline. The methods and results of the two scoping reviews are shown in the manuscript describing the framework and its Supplementary Materials (1). Second, the framework was drafted based on the concepts and then refined in iterative brainstorming sessions and subsequent discussions with representatives from various stakeholder groups, including academia and representatives of HTA agencies and patient associations.

The IHTAM framework defines a general innovation process with three phases (i.e., “Identification,” “Development,” and “Implementation”) and nine subphases (e.g., “Design Prototypes” and “Plan for Implementation”). Also, the framework illustrates how stakeholders could be involved by clarifying the three roles they can play (i.e., “Developers,” “Practitioners,” and “Beneficiaries”). Although the IHTAM framework was developed, the framework applicability in the innovation practice of HTA methods has not been evaluated.

Hence, the aim of this study was to explore applicability of the IHTAM framework in three cases of development of quantitative methods and to improve its applicability by updating the framework. This research was performed as part of the HTx project.

## Methods

### Case description

The cases were identified from the HTx project, which has received funding from the European Union’s Horizon 2020 research and innovation programme under grant agreement No 825162 (12). The HTx project consists of four case studies (CS1, CS2, CS3, and CS4), but here, we only discuss the application of the framework in three of them. In CS4, only one HTA stakeholder group (i.e., researchers) engaged in the method development, and there was no plan for implementing this method during the HTx project. Therefore, applying the framework to CS4 was deemed inappropriate. Descriptions of the three cases are shown in Table 1.

**Table 1.** Three cases in which the IHTAM framework was applied

Case	Method	Population	Intervention	Outcome
1	Models to evaluate cost-effectiveness of NTCP models^a^	Patients with head and neck cancer	Proton therapy	Radiation-induced complications
2	Prognostic risk prediction models	Patients with type–1 or type–2 diabetes	All types^b^	Diabetic complications^c^
3	Methods to use real-world data in health technology assessment settings (i.e., TTE, LTMLE, and CML)^d^	All types	All types	All types

*Note: The NTCP model indicates the normal tissue complication probability model.*

a NTCP models are models used in the field of radiotherapy to estimate the risk (i.e., probability of occurring) of radiation-induced complications (13).

b All types indicate that the method(s) in a case can be applied to any population, intervention, or outcome.

c Diabetic complications refer to macrovascular complications (e.g., coronary heart disease), microvascular complications (e.g., diabetic renal disease), and short-term complications (e.g., hyperglycemia).

d TTE is a method to apply the study design principles of randomized trials to observational studies that aim to estimate the causal effect of an intervention (14), LTMLE is a method to estimate the causal effects using observational data (15), CML is a machine learning model that involves the process of identifying causal inference (16). These methods were not readily applicable to health technology assessment due to quality concerns (e.g., time-varying confounding) (17;18).

CML, causal machine learning; LTMLE, longitudinal targeted maximum likelihood estimation; TTE, target trial emulation.

### Application of the IHTAM framework

Before applying the framework, all case study leaders were asked to assess the feasibility of evaluating the framework applicability in their own case. Since feedback would be collected from all case study members, a case that hardly represented HTA stakeholders in practice (e.g., only one HTA stakeholder group) might be excluded. An overview of the IHTAM framework is shown in Supplementary Material S1. According to the framework (1), case study leaders were recommended to apply the framework in four steps, which are shown in Table 2. To ensure the case study leaders understood how to apply the framework, we followed up the framework application progress in each case, and provided assistance in three steps.

**Table 2.** Four steps to apply the IHTAM framework

Step 1	To consider all (sub)phases and three innovation roles (i.e., developer, practitioner, and beneficiary) within the IHTAM framework, and to judge their relevance to the case
Step 2	To discuss whether new (sub)phases or roles of innovation apply
Step 3	To consider challenges of innovation
Step 4	To facilitate collaboration by inviting HTA stakeholders from multiple backgrounds (e.g., patients and industry), based on the case-specific needs

In the beginning, we organized a face-to-face training session for case study leaders and consortium members during the HTx project consortium meeting, in April 2022. During the training session, one researcher (LJ) introduced the structure of the IHTAM framework, and explained how to apply the framework, using the patient-reported-outcome-measures toolbox, co-developed earlier as part of the HTx project, as an example method (19). Any confusion from stakeholders was solved through questions and answers.

Next, we followed up each case, by attending the regular meetings, which were held approximately every 2 months for each case. In each meeting, at least one researcher (JW or LJ) attended, reminded case study leaders to keep applying the IHTAM framework, and answered relevant questions. The follow-up lasted for 1 year.

At the end of the follow-up, case study leaders were asked to systematically report the method innovation progress, using the IHTAM framework. Each case was given 30 min during the face-to-face general assembly meeting of the HTx project, in May 2023. Before the general assembly meeting, we provided case study leaders with a slide template, and resolved any outstanding questions through e-mail or an online meeting.

### Evaluation and improvement of the framework applicability

After the general assembly meeting in 2023, we invited case study leaders and consortium members to provide feedback on the applicability of the IHTAM framework, through an online survey or interview, based on the invitees’ preference. The online survey or interview involved two open questions: first, which aspect of the IHTAM framework could improve the understanding of progress made in case studies, and second, which aspect of the IHTAM framework did not improve the understanding and could be further improved. All feedback was recorded by one researcher (LJ), and then independently summarized by two researchers (LJ and JV) using NVIVO12. Any discrepancy was solved through discussion.

Based on the feedback, we updated the IHTAM framework to improve its applicability. The updated version was first prepared by authors, then edited by case study leaders and consortium members, and finalized by four authors (LJ, JV, AM, and WG) in a group meeting.

## Results

### Framework application to case studies

**Figure 1.**
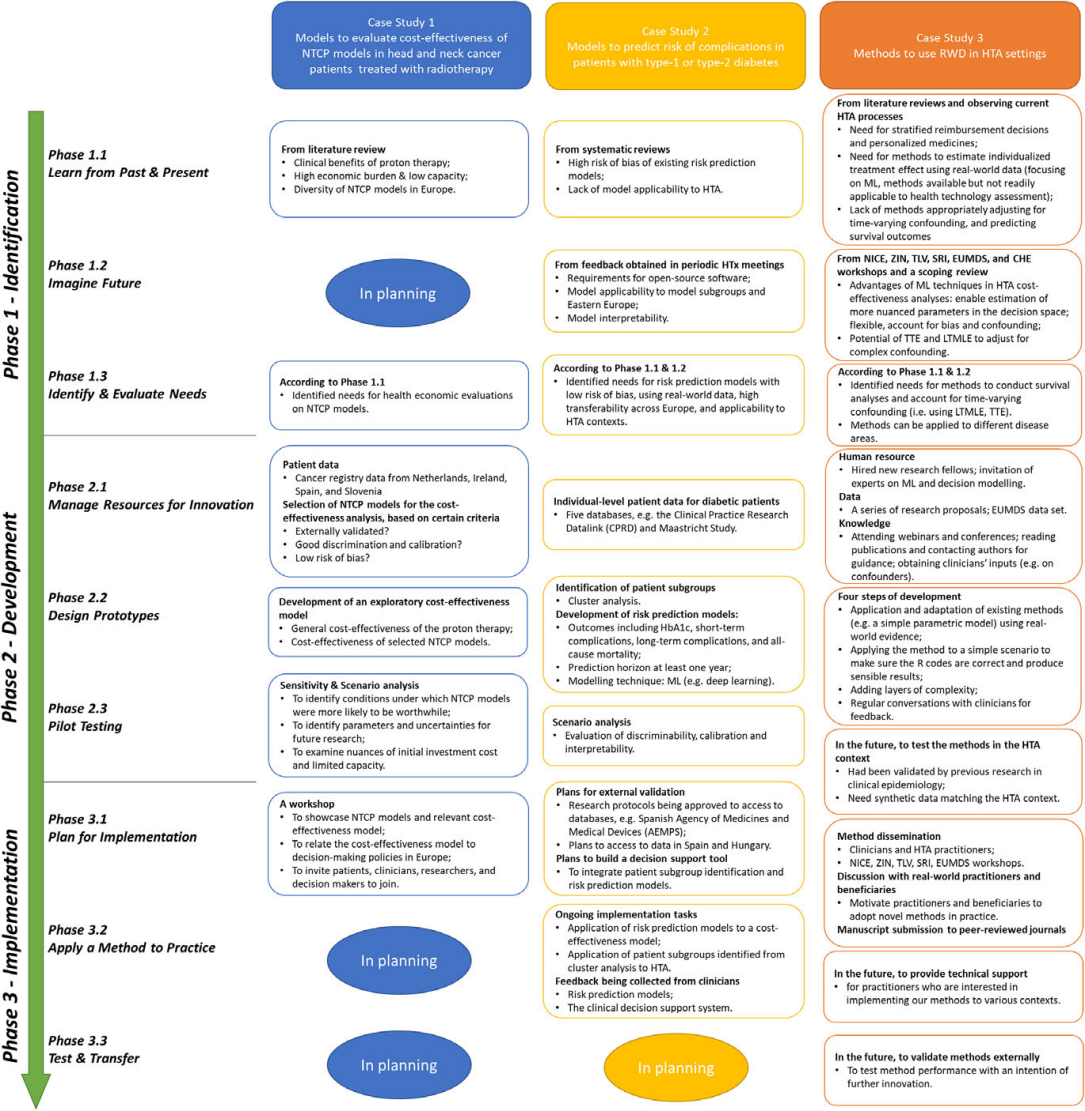
General innovation progress of HTA methods, reported by leaders of the three case studies. ML indicates machine learning; NTCP, normal tissue complication probability; RWD, real-world data; HTA, health technology assessment; TTE, target trial emulation; LTMLE, longitudinal targeted maximum likelihood estimation; NICE, National Institute for Health and Care Excellence (England); ZIN, National Health Care Institute (Netherlands); TLV, Dental and Pharmaceutical Benefits Agency (Sweden); SRI, Syreon Research Institute (Hungary); CHE, Center for Health Economics (University of York); EUMDS, European Myelodysplastic Syndromes Registry.

After the 1-year follow-up (about six periodic meetings for each case), three case study leaders, who were researchers, and 24 consortium members, from research institutes (n = 19), HTA agencies (n = 4), and patient organizations (n = 1) attended the general assembly meeting. The general progress of HTA method innovation, reported by case study leaders, is shown in Figure 1. The roles (i.e., developers, practitioners, and beneficiaries) of case study member, with some examples, which were reported by case study leaders are shown in Supplementary Material S2. In summary, none of the case studies completed all (sub)phases of the “Implementation” phase, but one or two subphases (e.g., “Apply a Method to Practice”) were in planning by at least one of the case studies. CS3 made the plans for the subphases, but they had yet to be conducted. Also, all the three roles (i.e., developers, practitioners, and beneficiaries) have been played by members of all the three cases.

#### The “Identification” phase

Some gaps in the HTA field and limitations of existing methods were identified, from literature reviews (all cases) or by observing practice in HTA settings (CS3). For example, in CS1, the proton therapy had clinical benefits, but its high economic burden and low capacity restricted its access to patients with head and neck cancer. Although the NTCP models used to select patients for proton therapies were available, information was lacking on the cost-effectiveness of these models. In the subphase “Imagine Future,” scenarios on what future HTA processes may look like were identified through feedback obtained during periodic HTx meetings (CS2) or workshops of HTA agencies plus a scoping review (CS3). In contrast, in CS1, a plan was made to identify future scenarios for using NTCP models for treatment and reimbursement decision-making. According to the previous two subphases, the needs of the novel HTA method(s) were identified in the three cases.

#### The “Development” phase

Key resources needed for developing a method were gathered in all the three cases (subphase “Manage Resources for Innovation”). More specifically, all cases involved the collection of data used for method development (e.g., cancer registry data in CS1), while CS1 and CS3 reported case-specific resources. For example, in CS3, experts in the fields of machine learning and decision modeling, a case-specific human resource, were invited to aid with method development. After resource management, all cases involved a case-specific process of developing method prototypes. For example, CS2 involved cluster analyses and development of risk prediction models using machine learning techniques, and CS3 needed clinicians’ inputs for method development. In the subphase “Pilot testing,” sensitivity or scenario analyses were conducted in CS1 and CS2, to investigate method uncertainty or performance. In contrast, a plan was made in CS3 on this subphase, and data matching some HTA contexts would be used to test the methods in the future.

#### *The*” *Implementation” phase*

All the cases involved a case-specific process of planning for implementation. In CS1, a workshop was organized to disseminate the method (i.e., a cost-effectiveness model), and to explain how the method was linked to the HTA decision-making policy in Europe. In CS3, several workshops were organized to not only disseminate the methods but also understand the motivations of potential case-specific practitioners (e.g., HTA agencies) or beneficiaries (e.g., clinicians) to adopt the methods. In contrast, method dissemination in CS2 was conducted through developing a tool (i.e., decision-support tool) for potential model users (e.g., clinicians). Additionally, a plan of model external validation was made in CS2 to investigate model transferability across countries. In the subphase “Apply a Method to Practice,” only CS2 reported ongoing tasks, as risk prediction models were being incorporated into a cost-effectiveness model, and relevant patient subgroups were being applied to HTA cluster analyses. Although CS3 involved no ongoing task in this subphase, a plan was made by developers to provide technical assistance to future practitioners who feel interested. Finally, no case involved ongoing tasks related to the subphase “Test & Transfer,” which involved testing method performance during implementation with the intention of further innovation. Although all case studies had not yet entered this subphase, leaders of CS3 considered it relevant and planned for externally validating methods they developed.

### Evaluation of the IHTAM framework applicability

Of the 28 attendees of the general assembly meeting in May 2023, when the case progresses were reported using the IHTAM framework, 3 were authors who collected feedback and updated the framework, 7 were case study leaders, and 18 were consortium members. Two case study leaders (labeled as L1-L2) and 12 consortium members (labeled as A1-A12) provided feedback. Of those without feedback, only one consortium member mentioned the reason: “did not realize that case study leaders tried to use the IHTAM framework,” so the framework could not be judged. In summary, all case study leaders and most consortium members (n = 8) who provided feedback stated that the IHTAM framework had improved their understanding of HTA methods innovation. Meanwhile, all case study leaders and most consortium members (n = 7) pointed out current limitations of the framework and provided suggestions on how to address them. The summarized feedback on the framework applicability is shown in Table 3, while all detailed feedback is available upon reasonable request.

**Table 3.** Feedback regarding the IHTAM framework applicability

Feedback	Number of stakeholders who provided feedback (2 CSL, 14 CM)	Example comments
Positive		
A structured way of thinking	2 CSL + 6 CM	“The framework helps to structure discussions about the case studies,” “standardizes our ontology,” and shows “how the next element in a study builds upon the previously completed tasks.”
Relevant to the innovation process of case studies	2 CSL + 5 CM	“Stakeholders could obtain many details on the needs for an HTA method and how it was developed”; The Implementation phase is “practical,” as it evaluates “where is a capacity to apply methods” and “where the healthcare system can benefit from it.”
Could improve multi-multidisciplinary collaboration	2 CSL + 5 CM	The framework “could be understood by stakeholders without any HTA background” and reminds stakeholders that “innovative methods need more collaborative efforts.”
Negative		
Need a loop structure	4 CM	The framework could include a “spiral” structure to include “long learning circles” of innovation, and it was not necessary to “move to clockwise direction.”
User-friendliness to be improved	1 CSL + 1 CM	“The framework may be more easily used, if it includes a tool (e.g., checkbox). For example, when users use the tool, they could know what has been done, and what could be done in the future.”
Risk of misinterpretation	1 CSL + 4 CM	Stakeholders “might have different understanding of what each step means to them,” and could “misinterpret” some of these steps, e.g., whether “‘Design Prototypes’ includes modeling.”
Some IHTAM subphases less applicable	2 CSL + 5 CM	“Some steps do not apply when we apply existing methods, e.g., ‘Design Prototypes’”; “‘Pilot testing’ is hard to follow in the case of developing risk prediction models”; “The third phase ‘Implementation’ is less easy to follow, as practitioners mainly use guidelines, either developed by the center where they work or scientific societies, to proceed with” method innovation.

CM, consortium member; CSL, case study leader; HTA, health technology assessment.

The improved understanding of consortium members could mainly be summarized into three points. More specifically, the framework provided a structured way of thinking, was relevant to the innovation process of case studies, and could improve multi-multidisciplinary collaboration. In contrast, the framework has four main limitations, including lack of a loop structure, lack of a checkbox (template, checklist, etc.) attached to the conceptual framework for increasing user-friendliness, risk of misinterpretation on meanings of a framework (sub)phase, and irrelevance of several IHTAM subphases (e.g., “Design prototypes”) to some case studies.

## A roadmap to complement the conceptual framework

A roadmap was designed to complement the IHTAM framework, taking the identified limitations into account. The roadmap is shown in Table 4, and a flow diagram illustrating the use of the roadmap is shown in Supplementary Material S3. The roadmap has three main features. First, it includes 48 items that covers all content of the original conceptual framework. With the roadmap, HTA stakeholders can more easily know what has been done and what to do next, by simply comparing roadmap items with their actions. Second, the roadmap includes two types of items, which interrelate with each other. Action items show what actions of innovation may need to be done, while Reporting items show issues to be reported to the audience (i.e., Reporting items). For example, in Table 4, the Action item A1.1.1 can inform stakeholders about the necessity of and the approach to identifying limitations of an existing HTA method, while the Reporting item R1.1.1 can remind stakeholders of reporting relevant actions (e.g., techniques used to identify the limitations). With the two items, some issues around misinterpretation can be solved, as sufficient reporting of actions can help avoid misunderstandings of stakeholders from various knowledge backgrounds. The third feature of the roadmap is a loop structure, which enables the design of a case-specific innovation process. Under each Action item, stakeholders are asked to judge whether the action has been taken in their case. Based on the judgment, the roadmap leads stakeholders to different items. With the loops, (sub)phases or actions considered irrelevant to a case can be skipped, while those considered relevant can even be repeatedly conducted.

**Table 4.** The IHTAM roadmap

Actions for innovating an HTA method	Reporting a process of innovation
*Identification phase——1.1—Learn from past and present*	
A1.1.1 Has the research technique(s) (e.g., surveys, interviews, literature review) been used to identify limitations of an existing HTA method? Go to R1.1.1 and A1.1.2.	R1.1.1 If Yes Report the research technique(s) used. If No Specify the reason why the research technique(s) is not used.
A1.1.2 Have all relevant HTA stakeholder groups been involved in identifying limitations of existing HTA methods? Go to R1.1.2 and A1.1.3.	R1.1.2 If Yes or No Report HTA stakeholders involved (e.g., HTA agencies, clinicians, patients, etc.). If No Specify how the lack of stakeholder groups could influence identification of needs of a novel HTA method.
A1.1.3 Have limitations of existing HTA methods been identified? If Yes, go to R1.1.3 and A1.2.1. If No, go to A1.2.1.	R1.1.3 If Yes List the identified limitations of existing HTA methods; If available, specify the heterogeneity of limitations identified by different HTA stakeholders.
*Identification phase—1.2—Imagine future*	
A1.2.1 Have scenarios on what the future HTA process may look like been imagined (e.g., through brainstorming techniques)? If Yes, go to R1.2.1 and A1.2.2. If No, go to A1.3.1.	R1.2.1 If Yes Specify the technique(s) used to imagine future scenarios; Describe what future scenarios may look like. If No Specify the reason why future scenarios is not imagined.
A1.2.2 Have enablers and barriers to reach future scenarios been outlined? If Yes, go to R1.2.2 and A1.3.1. If No, go to A1.3.1.	R1.2.2 If Yes List the technique(s) used to outline enablers and barriers, such as interviews and surveys; List enablers and barriers to reach future scenarios; Propose potential solutions to utilize facilitators and to overcome barriers.
*Identification phase—1.3—Identify and evaluate needs*	
A1.3.1 Based on Phases 1.1 and 1.2, has need(s) for a novel HTA method been identified? If Yes, go to R1.3.1 and A1.3.2. If No, go to A3.1.1, to plan to implement an existing method.	R1.3.1 If Yes Define the need(s) to be satisfied by an HTA method; According to R1.1.3 & R1.2.1 & R1.2.2, summarize how the needs are identified.
A1.3.2 Does the need(s) vary across contexts (e.g., different types of health technologies, disease areas, geographic areas, etc.)? Go to R1.3.2 and A1.3.3.	R1.3.2 If Yes or No Define the context(s) where a novel HTA method is needed. If No Specify why the need(s) can be similar across contexts.
A1.3.3 Is it necessary to develop an HTA method, or is transforming an existing method sufficient? If developing an HTA method is necessary, go to R1.3.3 and A2.1.1. If not necessary, go to R1.3.3 and A3.1.1, to evaluate transferability of an existing method.	R1.3.3 If Yes or No Specify the rationale to develop an HTA method or to transform an existing method.
*Development phase—2.1—Manage resources for innovation*	
A2.1.1 Are resources sufficient to develop or transform an HTA method? If Yes, go to R2.1.1 and A2.1.2. If No, go to R2.1.1 and A1.1.1.	R2.1.1 If Yes Report the technique(s) used to evaluate the resource sufficiency; list all resources needed to develop or transform an HTA method, such as time, finance, and knowledge. If No Specify why resources are not sufficient.
A2.1.2 Is it necessary to set priorities for needs (e.g., those identified from various contexts) that a method addresses, given limited resources? Go to R2.1.2 and A2.1.3.	R2.1.2 If Yes or No Specify the reason why setting priorities is (not) necessary. If Yes Report the priority list for needs; Specify the rationale for the priority.
A2.1.3 Have HTA stakeholders other than researchers been invited for method development or transformation? Go to R2.1.3 and A2.2.1.	R2.1.3 If Yes or No List all HTA stakeholders involved in developing or transforming an HTA method. If No Specify the reasons why stakeholders other than researchers are not invited.
*Development phase—2.2—Design prototypes*	
A2.2.1 Have a method prototype and its derivative versions been developed or transformed, based on the heterogeneity of needs across contexts? Go to R2.2.1 and A2.2.2.	R2.2.1 If Yes Specify how the versions of a method prototype could address the heterogeneous needs. If No Specify the reason why the heterogeneity of needs are not considered.
A2.2.2 Has the ease of implementing the method in practice been considered, when developing or transforming a method prototype? Go to R2.2.2 and A2.3.1.	R2.2.2 If Yes Specify how the ease of implementation was considered in the method prototype. If No Specify how it could impact the method implementation.
*Development Phase—2.3—Pilot Testing*	
A2.3.1 Have pilot case studies been conducted to test validity of the method prototype and its derivative versions? Go to R2.3.1 and A2.3.2.	R2.3.1 If Yes Describe case studies conducted and HTA stakeholders involved; Evaluate how the case studies could simulate real-world practice; Report validity of the method prototype and its derivative versions. If No Specify how it could impact the method validity.
A2.3.2 Has applicability to other HTA contexts been taken account. When developing or transforming the method? Go to R2.3.2 and A2.3.3.	R2.3.2 If Yes Report techniques used to evaluate transferability (e.g., interviews with practitioners); List all HTA stakeholders who evaluate transferability, and report contexts they belong to (e.g., geographic and therapeutic areas). If No Discuss how it could impact transferability of the method.
A2.3.3 Can the HTA method be disseminated and implemented in various contexts, considering the validity, applicability, and transferability of the method prototype and its derivative versions? If Yes, go to R2.3.3 and A3.1.1. If No, go to R2.3.3 and A2.1.1, for another round of method development.	R2.3.3 If Yes or No Report the process on how the decision on dissemination and implementation is made; Describe contexts where an HTA method is disseminated or implemented; Specify potential causes of preventing dissemination and implementation (e.g., design flaws, lack of transferability, or wrong operations).
*Implementation phase—3.1—Plan for implementation*	
A3.1.1 Has the HTA method been disseminated or diffused? Go to R3.1.1 and A3.1.2.	R3.1.1 If Yes Report approaches used for dissemination (e.g., training practitioners) or diffusion (e.g., scientific publications); Report the involvement of developers, practitioners, and beneficiaries in the action of dissemination. If No Specify the reason.
A3.1.2 Has an implementation strategy been developed, for guiding the resources needed for conducting and monitoring the implementation, and for motivating potential practitioners to adopt the novel HTA method? Go to R3.1.2 and A3.2.1.	R3.1.2 If Yes Report the implementation strategy; Report resource needed for conducting and monitoring the implementation; Report planned approaches used to motivate practitioners in real-world practice. If No Describe the impact of method implementation without an implementation strategy.
*Implementation phase—3.2—Apply a method to practice*	
A3.2.1 Is technical assistance available from developers during method implementation in real-world practice? Go to R3.2.1 and A3.2.2.	R3.2.1 If Yes Describe the ways developers can be approached; Describe what type of technical assistance developers can provide. If No Specify the reason, and evaluate the impact
A3.2.2 Is the method implementation continuously monitored by developers, and is feedback from practitioners and beneficiaries accessible to developers? If Yes, go to R3.2.2 and A3.2.3. If No, go to A3.2.3.	R3.2.2 If Yes Describe how method implementation is monitored in practice; Report the technique(s) used to obtain feedback; Summarize feedback. If No Specify the reason.
A3.2.3 Have implementation strategies been adjusted during implementation? Go to R3.2.3 and A3.3.1.	R3.2.3 If Yes If available, report reasons why implementation strategies are adjusted (e.g., tailored contexts); Describe the adjusted implementation strategies; Describe impact of the adjusted implementation strategies. If No Specify the reason.
*Implementation phase—3.3—Test and transfer*	
A3.3.1 Have information on validity of an HTA method been obtained from various contexts during method implementation? Go to R3.3.1 and A3.3.2.	R3.3.1 If Yes Report validity of the method; Report contexts where information on method validity are obtained. If No Specify the reason.
A3.3.2 Have information on adoption of an HTA method been obtained from various contexts, during method implementation? Go to R3.3.2 and A3.3.3.	R3.3.2 If Yes Report the extent to which practitioners and beneficiaries adopt the method; Report contexts where relevant information are obtained. If No Specify the reason.
A3.3.3 According to R1.3.3 and R3.3.1 and R3.3.2, could the method be directly applied to all contexts of interest? If Yes, go to R3.3.3 and A1.1.1, for another round of identifying limitations of existing HTA methods. If No, go to R3.3.3 and A2.1.1.	R3.3.3 If Yes Specify the reason. If No Describe contexts where a method could be directly applied; Describe contexts where a method could not be directly applied; Specify facilitators and barriers of transferring the method.

*Note: A indicates Action items, and R indicates Reporting items. Action items show what actions of innovation may need to be done, while Reporting items show issues to be reported to the audience.*

## Discussion

In this study, we applied the IHTAM framework to three case studies in the HTx project, which were relevant to innovating a cost-effectiveness model, risk prediction models, and approaches to exploiting real-world evidence in HTA settings. The IHTAM framework was in general appreciated in the three case studies, as it provided a structured way of thinking, is highly relevant to the innovation process of case studies, and it could improve multidisciplinary collaboration. Based on feedback from case study leaders and consortium members of the HTx project, who were informed on the reports of the three cases, we developed a roadmap that could complement the original conceptual framework by overcoming its limitations.

The IHTAM framework is the first conceptual framework that provides general guidance on how HTA methods should be innovated, and it can be used to develop or improve case-specific guidance on method development or implementation. For example, some guidelines, such as the Prediction model Risk Of Bias ASsessment Tool (20) and the Transparent Reporting of a multivariable prediction model for Individual Prognosis Or Diagnosis (21), have been developed for improving quality of risk prediction models. Although these guidelines proved to be robust (22;23), they did not provide much guidance regarding how to develop a model with wide HTA applicability, due to the lack of involvement of various groups of HTA stakeholders (24;25). The IHTAM framework, attached with the actionable roadmap, can be used to improve these guidelines. In one aspect, the IHTAM framework emphasizes the importance of identifying the needs for method innovation and evaluating the extent to which the needs are satisfied throughout the innovation process. Hence, this framework could help guideline developers raise awareness that the existing guidelines may not satisfy particular needs of the HTA stakeholders. In another aspect, the IHTAM framework could help guideline developers take transferability issues into account from the beginning of method innovation. As the needs are repeatedly emphasized, guideline developers could be motivated to build closer ties with the practitioners (e.g., modelers of a health economics model) and beneficiaries (e.g., HTA agencies). In addition, the IHTAM framework complemented by the roadmap could add value to HTA stakeholders who are involved in HTA method innovation. First, it facilitates knowledge transfer and exchange (KTE) among stakeholders with different knowledge backgrounds. KTE is an interactive process, and one of its primary purposes is to increase the likelihood that research evidence will be used in policy and practice decisions (25). In HTA settings, where HTA methods are applied to clinical or reimbursement decision-making, KTE is considered difficult as it involves a series of complex actions (18;26;27). Our roadmap may partly solve the complexity, as it can awaken the realization of a knowledge gap, between HTA stakeholders who are already involved in innovation and those who are not yet involved. For example, according to our roadmap, some items that need to be reported during method development include: how the versions of a method prototype could address the heterogeneous needs (Item R2.2.1), and how the ease of implementation was considered in the method prototype need to be specified (Item R2.2.2). Although developers are familiar with how methods they develop can be used, reporting such information to beneficiaries, who are not directly involved in method innovation but could benefit from a novel method, could enhance beneficiaries’ motivation. More specifically, beneficiaries, who are policy makers in some cases, may explore the added value of a novel method to HTA regulations in an early stage, and to provide in-time feedback that improves method transferability. For example, HTA bodies have been recommended to consider causal inference, when they develop guidance regarding HTA decision-making (27). Since ML methods (e.g., LTMLE) can be used to estimate the causal treatment effects directly, and may outperform traditional methods, such as propensity score matching (18), the applicability of these methods may be evaluated by HTA bodies. As shown in CS3 (Figure 1), with the IHTAM framework, developers have been encouraged to disseminate LTMLE in workshops, to motivate beneficiaries, and to make plans for providing technical support.

Another advantage of the roadmap is that it may motivate HTA stakeholders to participate in the action of method innovation. Given the quite long circle of a whole HTA method innovation process, a single stakeholder, regardless of the innovation role (e.g., developer), hardly participates in the whole process. As shown in our study, at the end of the 1-year follow-up, none of the three case studies went through all innovation (sub)phases, and only CS2 started to apply methods to practice. This long-circle challenge could be addressed by the updated framework. More specifically, by quantifying (sub)phases of the IHTAM framework into actionable items, stakeholders may know where they should take responsibility and when their roles may be taken over. For example, in CS2, some practitioners such as HTA modelers, applied the risk prediction models to a cost-effectiveness analysis. According to the IHTAM framework and roadmap, practitioners could describe how model developers are approached, and describe the types of assistance they need (e.g., how to load the risk prediction models in another software) (Item A3.2.1 & R3.2.1). Developers of risk prediction models could record feedback from practitioners after a cost-effectiveness model is developed (Item A3.2.2 and R3.2.2).

We recommend HTA stakeholders to use the roadmap in four steps. First, stakeholders may scan the IHTAM framework and the roadmap, to understand how to innovate an HTA method and how to involve other stakeholders in innovation. Second, stakeholders may understand the current innovation status of their cases and identify corresponding items in the roadmap as their starting point. The innovation does not necessarily start from identifying limitations of existing methods (i.e., Item A1.1.1), but it could start from any IHTAM (sub)phase of item. Moreover, an innovation process is not necessarily initiated by developers. For example, during the implementation of an existing method, practitioners may sense a lack of method transferability to a certain context. Then, they could start the innovation loop from the IHTAM subphase “Test & Transfer,” or the Item A3.3.1 of the roadmap. After evaluating the validity of methods during the innovation and method adoption of practitioners, they could make a decision on whether to initiate another round of identifying the limitations of existing HTA methods (Item A3.3.3 and A3.3.4). Once a starting point is identified, stakeholders may clarify their roles (e.g., developers, practitioners, and beneficiaries) and divide tasks accordingly. Moreover, an individual stakeholder may determine its stopping point, and if feasible, propose to stakeholders who to take over. One practical way of determining the stopping point is to draw a timeline for relevant tasks, based on task magnitude, and to assign the involved stakeholders.

Finally, it is recommended to build a log of innovation throughout an innovation process by following the IHTAM framework and its roadmap. The innovation log could record the actions of innovation and all relevant details. The innovation log could help stakeholders who participate afterward view the landscape and understand the details that are relevant to their roles. Current research projects, with a goal to innovate HTA methods, have already recorded innovation progresses in some ways. Still, with an innovation log, HTA stakeholders could take a step further, to link all relevant documents, and to help themselves figure out their roles in a big research project.

Our study has a number of limitations. One limitation is that only consortium members within the HTx project provided feedback regarding framework applicability, and only half of those responded. Another limitation is that more than half of the consortium members were more or less involved in at least one case study, so they had prior information (though this may be not complete) on the case studies before they responded to reports from case study leaders. The abovementioned limitations could cause an overestimation of model applicability. The main reason for including only stakeholders within the HTx project is the complexity of testing the applicability of a conceptual framework within a case study. In our research, a case involved development and implementation of an HTA method. Inviting a group of external stakeholders who jointly develop or implement a method for at least 1 year becomes less feasible. However, after the actionable roadmap is developed and the framework applicability can be evaluated beyond a case, external stakeholders with various backgrounds (e.g., payers and industry) can be invited to test the general framework applicability by giving suggestions on each roadmap item in the future. In our current research, the risk of subjectivity posed by internal stakeholders was minimized with the rigid coding technique. As two researchers independently summarized feedback, the obtained feedback, though maybe not complete, could objectively reflect the limitations of the conceptual framework approach. As the roadmap complementing the IHTAM framework was developed, we believe that the applicability of the updated framework is considerably improved. In addition, the stakeholders in our case studies might not adequately represent HTA stakeholders in practice. For example, CS2 only involves researchers and members of HTA agencies, while in practice, risk prediction models have impact on patients and healthcare professionals (28). Also, since the time required for innovating an HTA method can be longer than the duration of a research project, some (sub)phases of the IHTAM framework were not yet reached at the end of follow-up (Figure 1). Consequently, we could miss some potential suggestions for further improving the framework. Hence, we recommend future studies to test the framework applicability in various geographic and therapeutic settings where all HTA stakeholder groups potentially relevant to an HTA method are involved. Another limitation is that we only applied the IHTAM framework to three cases of quantitative methods, for example, models, rather than qualitative methods. However, we believe the stakeholder’s feedback on framework applicability, as well as the designed roadmap, is transferable to qualitative methods. One reason for this is that the IHTAM framework was originally developed based on actual innovation processes of qualitative methods, and the roadmap enables a method-specific innovation process.

## Conclusion

The IHTAM framework was generally applicable to case studies of innovating HTA methods. A roadmap and the conceptual framework approach could help facilitate knowledge transfer and exchange among HTA stakeholders with different knowledge backgrounds. Also, it could motivate stakeholders from understanding method innovation to action. To further examine the framework applicability, we recommend HTA stakeholders with various backgrounds (e.g., payers and industry) outside the HTx project to apply the framework to the innovation processes of different types of methods.

## Supporting information

Jiu et al. supplementary material Jiu et al. supplementary material

## Supplementary material

The supplementary material for this article can be found at http://doi.org/10.1017/S0266462324000564.
