# Supplementary material for: Roadmap to Innovation of HTA Methods (IHTAM): insights from three case studies of quantitative methods
Source: Int J Technol Assess Health Care. 2024 Nov 5;40(1):e49. doi: 10.1017/S0266462324000564 (PMC11563180; doi:10.1017/S0266462324000564)
Supplement: Jiu et al. supplementary material [file S0266462324000564sup001.docx]

**Appendix 1 Concept map of the IHTAM framework**

**
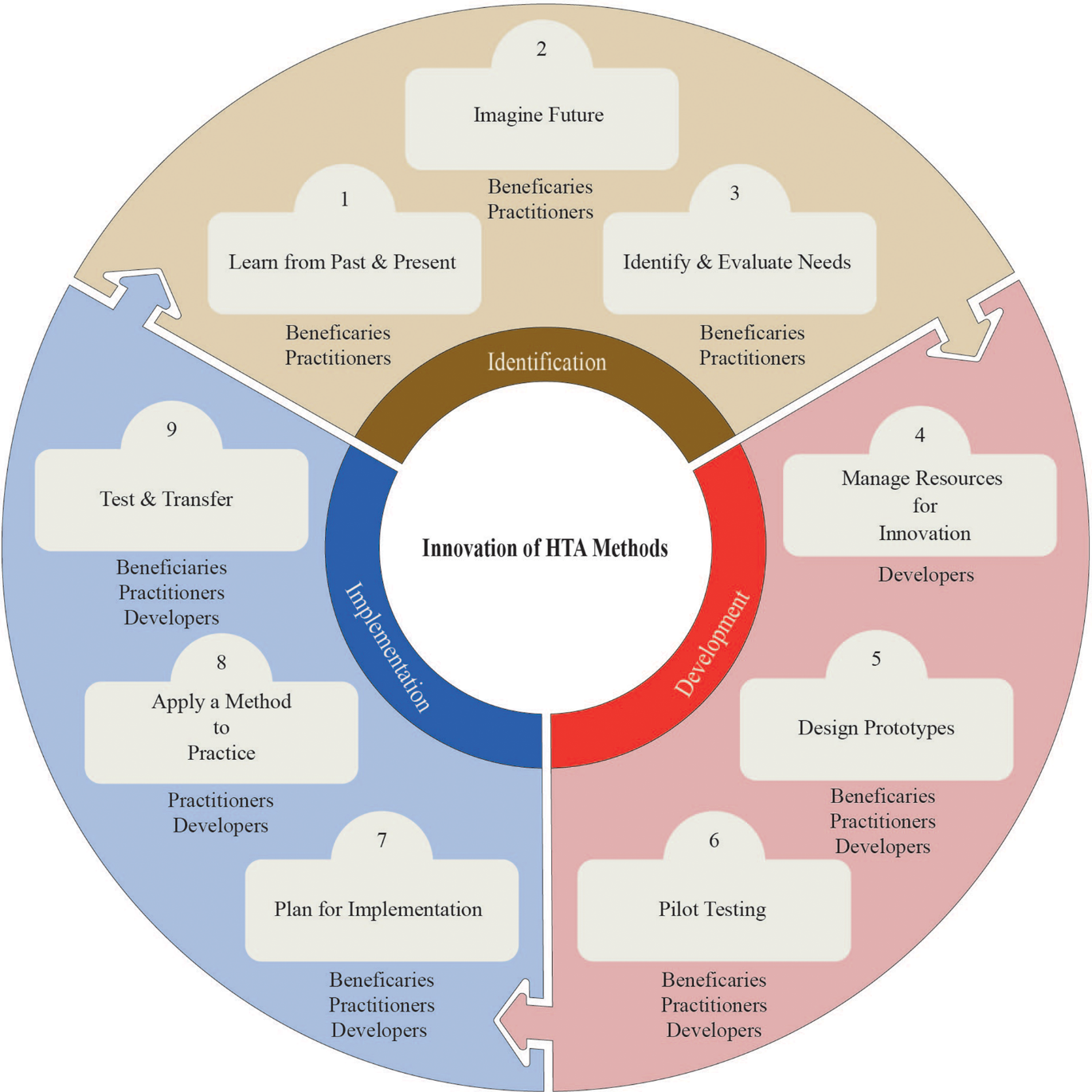
**

**Appendix 2 Examples on how HTA stakeholders participates in the three cases**

| **Who** | **HTA stakeholder group** | **Role** | **Example tasks** |
| --- | --- | --- | --- |
| **Case 1** | Researcher (n=3) | Developer | - Presentation - Technical assistance |
|  | Patient and clinician (NR) | Practitioner | - Application of the NTCP model using data from various countries for patient selection |
|  | HTA agency (n=1) | Beneficiary | - Application of the health economics models using data from various countries for HTA/reimbursement decision making |
| **Case 2** | Researcher (n=2) | Developer | - Identification of needs to implement patient subgroups and risk prediction models in a decision support tool |
|  | HTA agency (n=4) | Beneficiary | - Identification of outcomes of interest - Requirements about open source software - Explainability of methods - Interest of application to specific subgroups |
|  | HTA researcher (n=3) | Practitioner | - Expressed their needs for case study methods - Input in modelling (reviewed confounders / covariate lists) |
|  | Patient association (n=1) | Beneficiary | - Attended our presentations - Provided feedback - Expressed needs for case study methods - Evaluated how to tailor the methods to local contexts and whether the tailored method could be adopted. |
| **Case 3** | HTA researcher (n=3) | Developer | - Learned about limitations of current HTA processes - Identified available methods - Conducted literature reviews - Assessed what needs to be done for existing methods to be widely used in HTA. - Identified needs of stakeholders by presenting work and seeking help in constructing models |
|  | Clinician (n=4) | Developer | - Expressed their needs for case study methods - Input in modelling (reviewed confounders / covariate lists) |
|  | HTA researchers, Patients and HTA agencies (NR) | Practitioner and Beneficiary | - Attended presentations - Provided feedback during presentations - Expressed needs for case study methods - Evaluated how to tailor the methods to local contexts and whether the tailored method could be adopted |

*Note* NR indicate the number of stakeholders are not reported.

**Appendix 3 Flow diagram illustrating the use of the IHTAM roadmap**

**
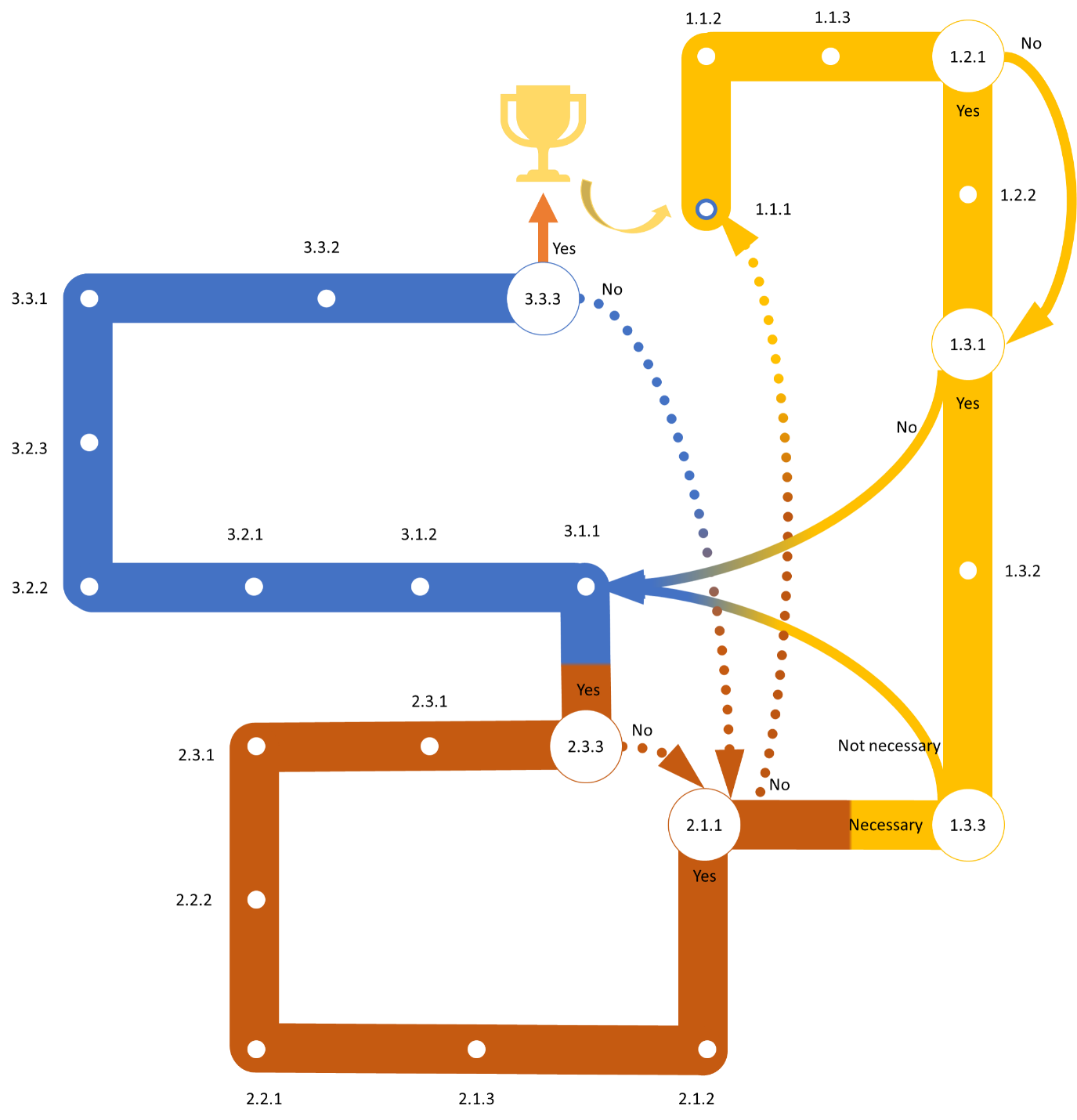
**

*Note* The three-digit numbers indicate the roadmap items: the first digit indicates the three innovation phases (i.e. “Identification”, “Development”, “Implementation”), which are colored in yellow, red, and blue, respectively; the second digit, the nine subphases (e.g. “Learn from past & present” and “Imagine future”); the third digit, the specific items of a (sub)phase. The hollow circles indicate the items linked to the loop structure: e.g., if needs for a novel HTA method is identified (Item 1.3.1), users may manage resources needed for method development (Item 1.3.2); otherwise, user may jump to Item 3.1.1, to plan for implementation of an existing method. The arrows indicate the loop structure: solid arrows, going forward; dashed arrows, going back.
